# A Novel Variant in Non-coding Region of *GJB1* Is Associated With X-Linked Charcot-Marie-Tooth Disease Type 1 and Transient CNS Symptoms

**DOI:** 10.3389/fneur.2019.00413

**Published:** 2019-04-24

**Authors:** Si Luo, Hui Jin, Jiajun Chen, Lei Zhang

**Affiliations:** Department of Neurology, China-Japan Union Hospital of Jilin University, Changchun, China

**Keywords:** Charcot-Marie-Tooth disease, *GJB1*, central nervous system manifestations, variant, pedigree

## Abstract

X-linked Charcot-Marie-Tooth disease type 1 (CMTX1) is a dominantly inherited peripheral neuropathy and is caused by mutations in gap junction beta 1 gene (*GJB1*). Here, a novel variant of c.-170T>G in *GJB1* was identified in a large Chinese CMTX1 pedigree. The proband presented transient “stroke-like” episodes in addition to the peripheral neuropathy. At the time of episode, he had transient hyperthyroidism. To our knowledge, this is the first variant found in non-coding region associated with transient central nervous system (CNS) symptoms and in this case, thyroid dysfunction might contribute to the episode. The mechanism of CMTX1 as well as the transient CNS symptoms waits to be elucidated.

## Introduction

X-linked Charcot-Marie-Tooth disease (CMTX) is an inherited axonal or mixed axonal-demyelinating disease of the peripheral nervous system, characterized by slowly progressive distal muscle weakness and atrophy, decreased or absent deep tendon reflexes, sensory abnormalities, and foot deformities (pes cavus and hammer toes). CMTX type 1 (CMTX1), occupying 90% of CMTX (1), is caused by mutations in gap junction beta 1 gene (*GJB1*) on chromosome Xq13.1. Apart from the neuromuscular manifestations, a small number of CMTX1 patients have transient central nervous system (CNS) symptoms and reversible cerebral white matter lesions (1) whose mechanisms are not completely understood (2). In this report, the clinical features of a big Chinese CMTX1 pedigree were described, in which the proband presented with transient CNS symptoms and transient hyperthyroidism. A novel NM_000166.5 c.-170T>G (chrX:70443032 GRCh37) variant in *GJB1* was found in this pedigree. To current reports, this is the first variant identified in non-coding region associated with transient CNS symptoms (3, 4) and thyroid dysfunction might be a contributor of this episode in this case.

## Case Presentation

**Figure 1:**
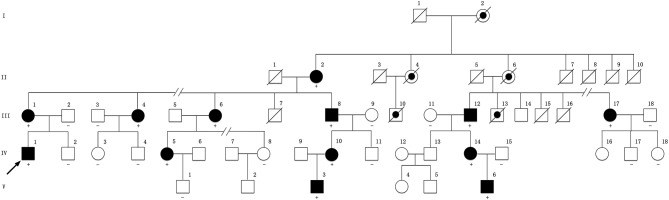
Pedigree of the family compiled with X-linked dominant inheritance. Square represents male and circle represents female. Filled symbol indicates affected. Dot symbol indicates deduced affected individual with medical history. A slash through a symbol indicates deceased individual. Arrow indicates the proband. The symbol “+” indicates a carrier of the variant. The symbol “–” indicates wild genotype. No symbol means the individual's genetic test is not available. The pedigree has been edited to maintain the confidentiality of family members.

The proband (IV-1 in Figure 1), a 28-year-old man, was admitted into our department for recurrent dysphonia and asymmetric weakness of four limbs with the right side more severely affected. He had experienced the similar episodes twice when he was 14 and 20 years old. The symptoms lasted 4–6 h and resolved without treatment. He denied any infection, exercises, or other possible inducer before the onsets. This time the symptoms completely disappeared after 5 h. During this episode, physical examination revealed bilateral facial palsy, dysarthria, and bilateral positive Babinski sign, with muscle strength grade 3 in the left limbs and grade 2 in the right limbs. After the episode, the neurologic examination showed normal muscle strength, slight intention tremor and unsteadiness when walking on a straight line as well as in the Romberg test. He also had high-arched feet and areflexia in all extremities.

Comprehensive infectious, metabolic, paraneoplastic, and inflammatory panels of the proband were negative. Serum potassium was normal. However, his free T_3_ (FT_3_) and free T_4_ (FT_4_) value were increased to 9.56 pmol/L (3.10–6.80 pmol/L) and 39.2 pmol/L (12.0–22.0 pmol/L), respectively. Meanwhile, the value of thyrotropin (TSH) was 0.006 mIU/L, much lower than the limit (0.372–4.94 mIU/L). Further, radioactive iodine uptake scan showed his iodine uptake rates were lower than normal and thyroid-specific autoantibody assays were all negative. Twenty days later, his FT_3_ and FT_4_ returned to normal. Five months after the episode, all thyroxine test results, including TSH, were all within the reference range and remained for the following 1 year.

**Figure 2:**
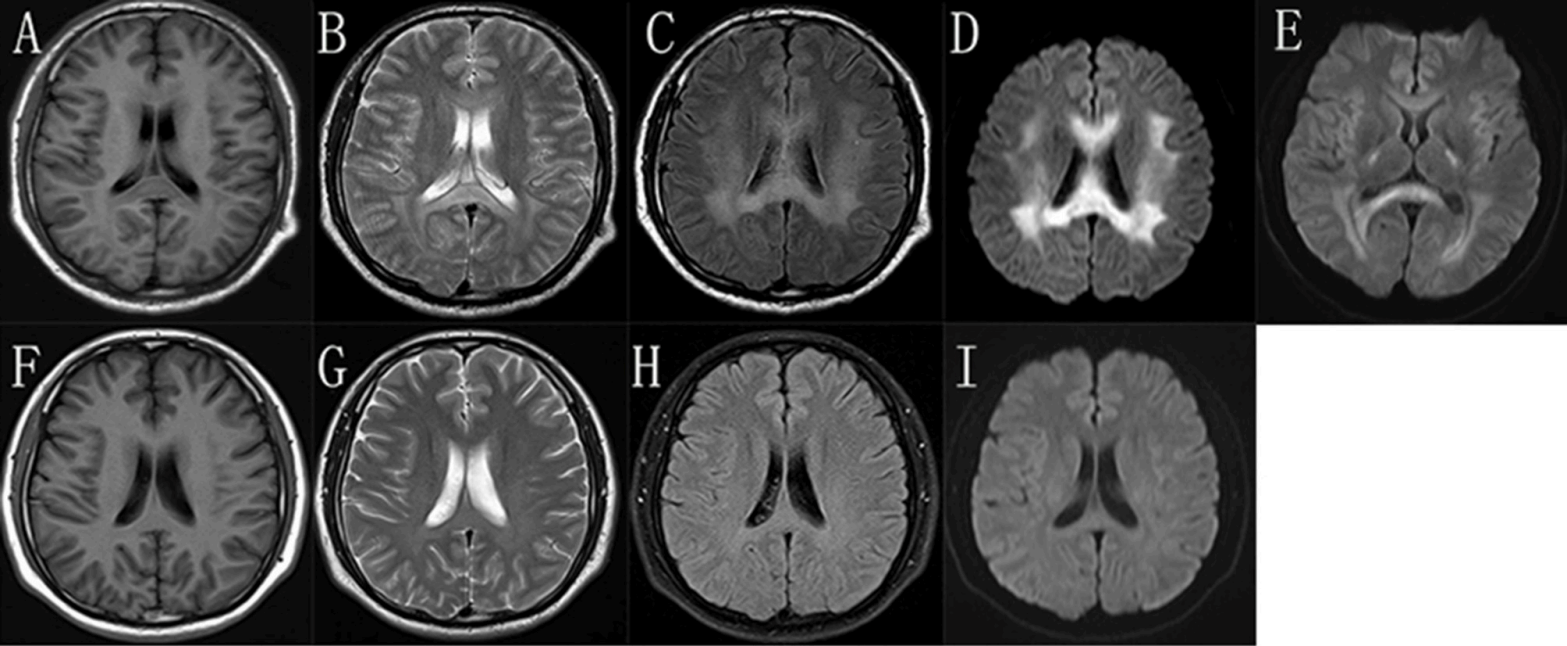
The brain MRI images of the proband. In the “stroke-like” episode, brain MRI showed slightly hypointensity in T1-weighted imaging **(A)** and slightly hyperintensity in T2-weighted imaging **(B)** in the bilateral deep white matter and the splenium of the corpus callosum. But there was obviously bilaterally symmetric hyperintensity in T2 FLAIR images and reduced diffusion in the same region **(C,D)**. MRI after 8 days showed improvement of the abnormal diffusion signal in white matter **(E)**. After 5 months, the images of T1-weighted **(F)**, T2-weighted **(G)**, T2 FLAIR **(H)**, and DWI **(I)** of his brain were almost normal.

During the episode, his brain MRI (Figures 2A–D) showed bilaterally symmetric abnormal T2 FLAIR hyperintensity in the deep white matter and the splenium of the corpus callosum (Figure 2C) and reduced diffusion (Figure 2D). The diffusion reduction disappeared mostly 8 days later (Figure 2E). Five months after the episode, the MRI of his brain were almost normal (Figures 2F–I). Electroneuromyography (EMG) showed reduced motor and sensory nerve conduction velocities, prolonged distal latency as well as reduced sensory and motor nerve amplitudes, indicating both demyelination and axon loss (S-Table 1). Specifically, his right/left median motor nerve conduction velocity is 33.6/37.7 m/s, comforming to the intermediate CMT (5). Brainstem auditory evoked potentials were normal.

**Figure 3:**
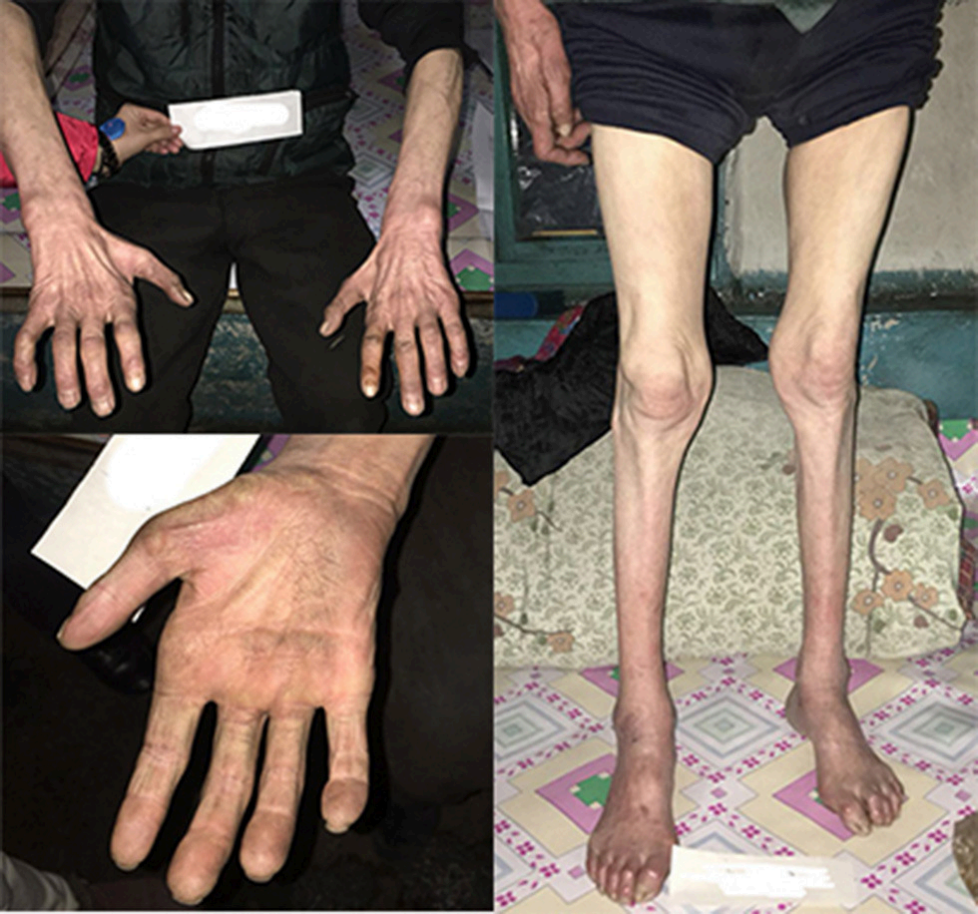
The pictures of III-8's limbs. He had atrophy of distal muscles below the knee and in the hands with cavus deformity.

This family is Chinese Muslim living in Jilin province. Pedigree analysis indicates an X-linked dominant inheritance (Figure 1). The proband is the only individual in the family who experienced “stroke-like” episodes. EMG was carried out to determine the affected in the pedigree. The common findings among affected males included difficulty running, distal weakness, pescavus, absent tendon reflexes, and atrophy of distal muscles with older affected more severely. III-8 presented typically with all the features above (Figure 3). Manifestations in female carriers were less severe and varied greatly. Some exhibited weakness and atrophy of hand muscles while some had lower limbs involved. However, a number of female carriers didn't show any symptom at all. The mother of the proband (III-1) was asymptomatic. However, examination displayed that she had high-arched feet and unsteadiness when walking on a straight line. EMG showed that she has demyelinating neuropathy with prominent axonal degeneration (S-Table 1).

**Figure 4:**
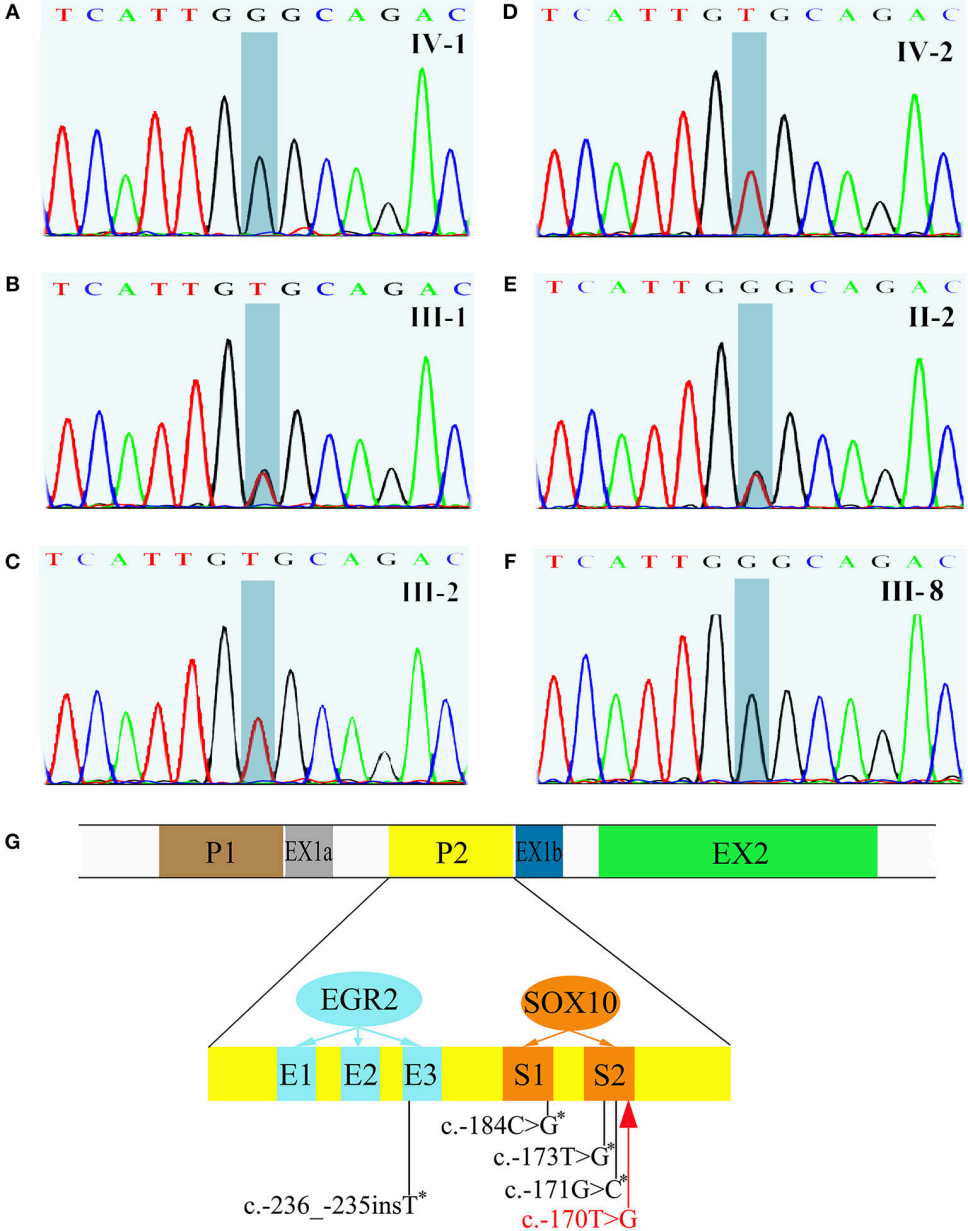
Sequencing results of *GJB1* and its schematic representation of the proximal Cx32 promoter region. **(A)** The proband had a hemizygous c.-170T>G variant. **(B)** III-1, the mother of the proband, had a heterozygous c.-170T>G. **(C,D)** III-1, the father of the proband and IV-2, the brother of the proband, were both wild genotype. **(E)** II-2, a 93-year old woman and the grandma of the proband without any clinical manifestation, carried a heterozygous c.-170T>G. **(F)** III-8, an affected male with typical neuromuscular manifestations had hemizygous c.-170T>G. **(G)** Schematic representation of the proximal Cx32 promoter region which indicates the positions of SOX10 (S1 and S2) and EGR2 (E1, E2, and E3) binding sites. *GJB1* has 2 tissue-specific promoters (P1 and P2) which are alternatively spliced. In neural tissue, *GJB1* transcription is driven via the nerve-specific promoter 2 (P2) upstream of non-coding exon 1b. Up to now, four mutations in the P2 region have been reported (indicated with asterisk). The c.-170T>G variant identified in this pedigree (red) neighbors c.-171G>C which has been proved impairing SOX10-mediated transcription of *GJB1*. This figure was modified from Neurology 2017;88:1445–1453. with permission.

All exons of *GJB1* of the proband were sequenced by Sanger sequencing. A novel hemizygous variant c.-170T>G was found (Figure 4A). It is located in the nerve-specific promoter P2 region of *GJB1*, neighboring c.-171G>C (designated as c.-146-25 G>C in the earlier edition of HGMD, Figure 4G), which has been shown reducing the expression of Cx32 (6). c.-170T>G cosegragated with the disease in this pedigree (Figure 1, Figures 4B–F) and was not present in 100 control DNA samples.

## Discussion

The clinical phenotype of CMTX1 is characterized by progressive muscle atrophy and weakness, areflexia, and variable sensory abnormalities. Electrophysiological and pathological studies of peripheral nerves showed the evidence of demyelinating neuropathy with prominent axonal degeneration. However, several patients also have manifestations of the CNS involvement or acute “stroke-like” symptoms (3, 7). These transient “stroke-like” symptoms usually have a childhood or adolescence onset and can present multiple neurological dysfunctions such as hemiparesis, paraparesis, monoparesis, ataxia, and dysarthria (7–10). In addition, respiratory distress (11), dysphagia (10), and disorientation (12) have also been reported. The episodes typically last for a few minutes to hours to days. Brain MRI during the acute phase typically presents increased signals in diffusion-weighted and T2-weighted sequences without the enhancement of gadolinium, involving the subcortical white matter and splenium of the corpus callosum. It usually takes months for these MRI changes to return to the baseline (7, 9, 10, 13–15). People have found these CNS manifestations do not correlate with the stage and severity of peripheral neuropathy. The patients in this pedigree compiled with the typical neuromuscular manifestations and the proband presented recurrent episodes of transient CNS symptoms, consistent with the features of CMTX1.

The protein of Cx32, encoded by *GJB1*, is a gap junction protein apportioned in the peripheral nervous system and CNS (16). Myelinating Schwann cells express Cx32, which provides a shorter pathway for the diffusion of small molecules and ions athwart the myelin sheath directly (17). Oligodendrocytes also express Cx32, which participates in the gap junction coupling with astrocytes (18). Mutations in non-coding DNA are considered a major cause of CMTX1 (4), but none has been related with transient CNS symptoms. The variant c.-170T>G, identified in this pedigree, is located in nerve-specific promoter P2 region of *GJB1*, where up to now, 4 mutations have been reported (Figure 4G) without benign variant found. Across a variety of species, the promoter P2 region is highly conserved. This variant we found is absent in 1,000 Genomes and dbSNP database and is not present in 100 control DNA samples. The phenotype of the patients is consistent with CMTX1 and this variant cosegregates with the disease in this big pedigree. So according to American College of Medical Genetics and Genomics standards and guidelines, this variant is classified as “likely pathogenic.” As to the pathogenic mechanism, since the neighboring c.-171G>C has been shown to impair SOX10-mediated transcription of *GJB1*, leading to a significant reduction in Cx32 expression (6), we speculated that c.-170T>G might cause the disease in the same way, but it waits verifying. On the other hand, the proband is the only patient with these CNS symptoms in the pedigree, although every patient in the family carries the variant. The reason might be the coexistence of another modifying pathogenic gene mutation. Expression studies of this variant in cultured cells and animal models are necessary to clarify the mechanism.

Transient CNS symptoms in CMTX1 patients have often been associated with metabolic stresses such as hyperventilation, febrile illnesses, infections, exercises, or high altitude (9, 10, 15, 19). In this case, a transient hyperthyroidism coexisted with this transient “stroke-like” episode of the proband. The CNS symptoms disappeared within several hours; the abnormality of brain MRI vanished mostly within several days; and hyperthyroidism gained remission in several months. It is possible that thyroid malfunction contributed to the incidence of CNS symptoms in this case and it needs more observation and reports from other doctors and researchers.

The mechanism by which *GJB1* mutations cause the transient CNS dysfunction in CMTX1 is still not clear. Paulson et al. argue that these CNS symptoms does not likely involve demyelination because they demonstrated increased magnetization transfer ratio, nor axonal degeneration because they were reversible over a short interval (9). But a detailed MRI examination using diffusion tensor imaging and magnetic resonance spectroscopy in a man with CMTX1 and transient CNS symptoms showed reversible reductions in fractional anisotropy and N-acetyl-aspartate levels, indicating reversible axonal damage associated with deficient oligodendrocyte gap junctions (20). The mutation in *GJB1* might disrupt the gap junction communication between oligodendrocytes and astrocytes, which leads to inability of these cells to regulate fluid exchange and finally cell edema. This can be proved by restricted diffusion in MRI (3, 7, 9). Some CMTX1 mutants associated with transient CNS events were expressed *in vitro* and their ability to form functional gap junctions was found reduced, thereby reducing the coupling between oligodendrocytes and astrocytes (2). At the same time, physiologic triggers, including metabolic stress, pro-inflammatory factors, and lower pH of the cerebrospinal fluid in the setting of altitudinal changes, may exacerbate this tenuous gap junction coupling and lead to the episodes of CNS symptoms (21–23). *In vivo*, neuroinflammation induced by lipopolysaccharide disrupted the main astrocyte-oligodendrocyte gap junctions, contributing to the increased sensitivity of Cx32 KO mice to lipopolysaccharide. The mutant of *GJB1* T55I, which was associated with transient CNS phenotypes, induced ER stress under inflammatory conditions, further exacerbating oligodendrocyte dysfunction and pathological changes in CNS (24). Elucidating the consequences of *GJB1* mutations both in peripheral nervous system and CNS is very important for the development of effective CMTX1 treatments, and waits more work.

## Conclusion

A novel *GJB1* variant of c.-170T>G in non-coding region was found in this big Chinese CMTX1 pedigree. This is the first report of variant in non-coding DNA sequence associated with transient CNS symptoms. Thyroid malfunction may contribute to the CNS symptoms in this case.

## Ethics Statement

This study has been reviewed and approved by the Ethics Committee of the China-Japan Union Hospital of Jilin University. Each member of the family provided written informed consent to the participation in the study, the genetic test, and authorized to publish the study including the photos in accordance with the Declaration of Helsinki.

## Author Contributions

SL and HJ were responsible for acquisition of data, drafting the manuscript. JC was responsible for revising the manuscript. LZ was responsible for study concept or design, drafting/revising the manuscript, and the final approval.

### Conflict of Interest Statement

The authors declare that the research was conducted in the absence of any commercial or financial relationships that could be construed as a potential conflict of interest.
