# Supplementary material for: A Novel Variant in Non-coding Region of GJB1 Is Associated With X-Linked Charcot-Marie-Tooth Disease Type 1 and Transient CNS Symptoms
Source: Front Neurol. 2019 Apr 24;10:413. doi: 10.3389/fneur.2019.00413 (PMC6491636; doi:10.3389/fneur.2019.00413)
Supplement: Supplementary file 1 [file Table_1.DOC]

**S-Table 1**

Electrophysiological characteristics of two patients in the pedigree

| Nerve | DL (ms) R/L | Amplitude(mV) R/L | CV (m/s) R/L | F wave (ms) R/L |
| --- | --- | --- | --- | --- |
| Patient IV-1 |  |  |  |  |
| Median motor | 11.4/11.2 | 6.8/9.3 | 33.6/37.7 | 39.8/36.4 |
| Ulnar motor | 12/10.6 | 6.5/7.1 | 22.2/39.7 | 41.4/40.8 |
| Tibial motor | 16.2/16.9 | 1.69/3.8 | 36.1/33.0 | － |
| Median sensory | 2.9/3.6 | 4.0/2.5 | 43.9/39.4 | － |
| Patient III-1 |  |  |  |  |
| Median motor | 7.4/9.1 | 7.6/5.4 | 43.9/42.7 | 27.4/21.8 |
| Ulnar motor | 8.4/8.5 | 7.7/8.7 | 46.0/47.1 | 27.0/31.0 |
| Tibial motor | 14.8/14.1 | 0.6/3.1 | 35.7/38.4 | 33.0/31.0 |
| Median sensory | 3.4/3.4 | 6.1/1.9 | 42.8/37.9 | － |
| Sural sensory | 3.3/2.7 | 9.4/8.6 | 36.4/37.5 | － |

R/L, right/left; DL, distal latency; CV, conduction velocity; NP= not presented
